# Tensions in perspectives on suicide prevention between men who have attempted suicide and their support networks: Secondary analysis of qualitative data

**DOI:** 10.1111/hex.12611

**Published:** 2017-08-14

**Authors:** Andrea S. Fogarty, Michael Spurrier, Michael J. Player, Kay Wilhelm, Erin L. Whittle, Fiona Shand, Helen Christensen, Judith Proudfoot

**Affiliations:** ^1^ Black Dog Institute and Faculty of Medicine University of New South Wales Sydney NSW Australia; ^2^ Faces in the Street St Vincent's Hospital St Vincent's Urban Mental Health Research Institute Sydney NSW Australia; ^3^ School of Psychiatry University of New South Wales Sydney NSW Australia

**Keywords:** behaviour, intervention, male, prevention, suicide, treatment

## Abstract

**Background:**

Men generally have higher rates of suicide, despite fewer overt indicators of risk. Differences in presentation and response suggest a need to better understand why suicide prevention is less effective for men.

**Objective:**

To explore the views of at‐risk men, friends and family about the tensions inherent in suicide prevention and to consider how prevention may be improved.

**Design:**

Secondary analysis of qualitative interview and focus group data, using thematic analysis techniques, alongside bracketing, construction and contextualisation.

**Setting and participants:**

A total of 35 men who had recently made a suicide attempt participated in interviews, and 47 family and friends of men who had made a suicide attempt took part in focus groups. Participants recounted their experiences with men's suicide attempts and associated interventions, and suggested ways in which suicide prevention may be improved.

**Results:**

Five tensions in perspectives emerged between men and their support networks, which complicated effective management of suicide risk: (i) respecting privacy vs monitoring risk, (ii) differentiating normal vs risky behaviour changes, (iii) familiarity vs anonymity in personal information disclosure, (iv) maintaining autonomy vs imposing constraints to limit risk, and (v) perceived need for vs failures of external support services.

**Conclusion:**

Tension between the different perspectives increased systemic stress, compounding problems and risk, thereby decreasing the effectiveness of detection of and interventions for men at risk of suicide. Suggested solutions included improving risk communication, reducing reliance on single source supports and increasing intervention flexibility in response to individual needs.

## 1 INTRODUCTION

Suicidal behaviour is associated with a range of individual, social and environmental risk factors including mental health diagnoses, severe depression symptoms, childhood adversities, family history of suicide, recent stressful life events, substance use, absence of social support and history of suicidal behaviour.^1^, ^2^, ^3^ It is therefore unlikely that there will be a simple explanatory model, and there are indeed few validated clinical models. However, the Interpersonal Psychological Theory of Suicide proposes specific psychological constructs which are supported by empirical data showing that suicidal ideation can be produced by the combination of “thwarted belongingness” and “perceived burdensomeness,” while suicide attempts are associated with the combination of “suicidal ideation,” a perceived hopelessness that the situation will change, and an “acquired capability for lethal self‐injury,” with the latter linked to pain tolerance and habituation to fear‐producing experiences.^4^

Against this backdrop gender differences are observed. Women exhibit higher rates of deliberate self‐harm,^5^ and suicidal ideation.^6^ Yet, men are more likely to die by suicide.^7^, ^8^ In Australia in 2015, there were 3027 suicide deaths, suicide was the leading cause of death among people aged 15‐44 years, and the rate of suicide in men was three times that of women.^9^ In 2007, 368 100 people reported suicidal ideation, and although women were more likely to have ever attempted suicide (6.5% vs 3.9%), the majority of suicides occurred in men.^10^

It has been proposed that women's behaviours have shaped the way suicidal presentations are perceived, resulting in lower rates of detection and intervention among suicidal men. However, men are also vulnerable to some common risk factors, with their presentations also framed by their specific sociocultural context.^6^ For example, men's experience of stressful events appears to be related to aspects of masculine identity.^11^ Stoic beliefs associated with avoidant, isolative coping strategies, involving affective or substance abuse issues, are more prevalent among men.^3^, ^12^ As a result, warning signs can take on more gender‐specific forms, such as increased aggression and substance abuse,^13^ yet may not be recognised as such.

In addition, men tend to select more violent means,^14^, ^15^ with a stronger association observed between acts of deliberate self‐harm and deaths from suicide,^6^, ^16^ as posited by the IPT. Similarly, only those with “acquired capability”—fearlessness, impulsivity, habituation to pain, violence and trauma—are likely to act on those thoughts,^17^ which is consistent with higher rates of death by suicide in men.

These findings point to the need to better understand the factors specific to suicide prevention in at‐risk males from the people involved, that is, the at‐risk men themselves and their friends and families. While suicide prevention programmes are available, there are questions about the effectiveness of such programmes over time^18^ and it is unclear whether there are specifically indicated approaches that are more suitable for men than women. Few suicide prevention programmes are tailored to men, and none have explored the complexities of effective suicide prevention from the perspective of at‐risk men, their friends and families. Such perspectives are critical to improve the ways in which people respond to suicidal crises, and the health system supports the needs of men who may not access services.

Our recently completed qualitative study of at‐risk men and their family/friends used thematic analysis^19^ to broadly examine the pathway to suicide attempts in men, as well as the protective factors, preventative strategies and strategies to interrupt acute and immediate risk specific to men.^11^ The goal of the current research was to more explicitly examine the specific tensions that interfere with effective suicide prevention. Thus, this study uses qualitative secondary analysis^20^, ^21^ and extends the broad findings of the first qualitative study, by examining: (i) the complexities of and tensions within suicide prevention approaches for at‐risk males, and (ii) considering these data, how suicide prevention and interventions for men may be improved.

To our knowledge, this is the first qualitative study of its kind involving both at‐risk men and their friends/families after a suicide attempt, in order to gain an in‐depth understanding of the complexities and divergent views involved in effective prevention of suicide in at‐risk males.

## 2 METHOD

### 2.1 Qualitative secondary analysis

The results are based on the principles of qualitative secondary analysis (QSA), which allows for “expanding understanding of a particular phenomenon”^22^ (p. 55) while simultaneously allowing for reduced participant burden and avoiding cost duplication. The purpose was to provide a more in‐depth understanding of the tensions that affect suicide prevention in men.

### 2.2 Participants

Two groups of participants were recruited in all states and territories across Australia: (i) adult men who had made a suicide attempt in the previous 6‐18 months, for a face‐to‐face interview, and (ii) adult family and friends of men who had made an attempt in the same time frame, for participation in focus group discussions. The 6‐ to 18‐month time frame was selected to minimise risk of triggering distress, while enabling detailed recall of experiences. To gain a breadth of views, family and friends were not necessarily related to the men interviewed.

The study was publicised through local, state and national mental health organisations, professional associations and community networks. Recruitment was extremely difficult, due in part to the stigma often accompanying suicidality, lack of awareness by some friends/family that a suicide attempt had taken place and, restrictions from the 6‐ to 18‐month time frame. Respondents were screened for suitability on the basis of current mental health and selection criteria, and those who participated were reimbursed $50AUD.

### 2.3 Measures

Prior to interviews and focus groups, participants completed the Patient Health Questionnaire‐9 (PHQ‐9)^23^ and the Generalized Anxiety Disorder 7‐item (GAD‐7),^24^ as well as demographic information and mental health history. The PHQ‐9 is a nine‐item self‐administered scale assessing current depressive symptoms, with total scores ranging from 0 to 27. An item on suicidal thinking is included. The seven‐item GAD‐7 measures severity of generalized anxiety by asking how often participants have experienced symptoms in the previous 2 weeks. Total scores range from 0 to 21. On both the PHQ‐9 and GAD‐7, total scores of 5, 10 and 15 represent mild, moderate and severe levels of depressive and anxiety symptoms, respectively. The measures were administered for the purposes of risk monitoring and management of any potential distress.

### 2.4 Procedure

Semi‐structured interview and focus group schedules consisting of open‐ended questions explored participants’ experiences with suicide attempts in men, including factors contributing to a suicide attempt, as well as perceived barriers to preventing suicide. All interviews and focus group discussions were conducted by a male member of the research team (MP) and lasted 45‐70 and 60‐90 minutes, respectively. With consent from participants, they were recorded and transcribed verbatim. No participants withdrew from the study.

### 2.5 Risk management

A risk management process was implemented throughout the project. Participants were monitored for signs of distress during the interviews/focus groups and were identified as “at risk” if they reported (i) a drop in mood after the interview/group discussion, which was assessed using a visual analogue scale where participants self‐rated how they were feeling before commencing and again at the close of the interview/focus group^11^; (ii) high levels of current distress, indicated by scores on the PHQ‐9; (iii) experiencing suicidal thoughts in the previous 2 weeks, indicated by a score of more than 0 on item 9 of the PHQ‐9; or (iv) distress during participation. Any participant who exhibited distress was followed up by the facilitator on the day, and a clinical psychologist was contracted in each location to provide follow‐up to any “at‐risk” participants in the following days. No participants required referral to clinical services.

As a standard part of the protocol, all participants received contact details for mental health services in their region and, in the week after participation, a phone call by the research team to check whether any distress had been experienced.

### 2.6 Data analysis

Digital recordings were transcribed, de‐identified and analysed using the NVivo qualitative data analysis software.^25^ During the initial thematic analysis, three interview and three focus group transcripts were selected by the interviewer and an independent member of the research team.^19^ First, the transcripts were openly coded, and concept patterns labelled. Next, the transcripts were cross‐coded and field notes were compared, to identify commonalities and differences. Once coding reliability was achieved, the two raters (MP, MS) separately coded the remaining transcripts. For the purposes of the current study, the coders used the line‐by‐line coding from the original analysis to identify the codes that related specifically to tensions in suicide prevention in the sample. Transcripts were re‐read, and in conjunction with the pre‐existing codes, five preliminary tensions in suicide prevention were identified. These preliminary tensions were used for the “construction” phase of analysis, which involved careful examination of the common elements and experiences of participants, such that recurring patterns or pathways of experience were identified. Once the five tensions were confirmed within the data, contextualisation through comparison and synthesis allowed for commenting on greater meaning across the individuals’ experiences.^22^

Several strategies were employed to reduce potential researcher bias, based on existing recommendations.^26^ The interviewer used open questions, and the data analysts kept notes while coding, using the participants’ language where possible to label and describe concepts. Weekly discussions were held to evaluate validity and usefulness of themes and to identify when data saturation occurred. Finally, the research team reviewed the findings to establish an acceptable, consensus map of the topic.^27^

The Human Research Ethics Committee of the University of New South Wales granted ethical approval for the study (HREC 13077).

## 3 RESULTS

### 3.1 Demographic and clinical profile

Thirty‐five men completed one‐to‐one interviews (median age 43 years, range 18‐67 years) and forty‐seven family and friends, including twenty‐six women (55%), participated in eight focus groups (median age 47 years, range 19‐65 years). About one‐third (34%) of men were currently employed, 46% were unable to work and 20% unemployed, studying or retired. Just over half (54%) had never married, 11% were currently married and 35% were separated or divorced. Approximately half of the family/friends were currently employed (53%), with 19% unable to work and 27% unemployed, studying or retired. About half (49%) were currently married or in a de facto relationship, 28% never married, and 23% separated, divorced or widowed. Men's and family/friends’ PHQ‐9 scores fell within the mild depression range (*M*=8.0, SD=6.2 and *M*=5.5, SD=5.7, respectively) and in the minimal to normal range on the GAD (*M*=6.3, SD=5.6 and *M*=4.1, SD=4.6, respectively). A majority of men (71%) and family/friends (87%) reported not experiencing suicidal ideation in the 2 weeks prior to interviews.

### 3.2 QSA results

Several tensions between men and their support network/care system were identified in the data. The tensions acted to increase problems, risk and stress, which in turn complicated or decreased the effectiveness of risk detection and intervention. Tensions related to five processes, four of which operated between individuals and their support system of family, friends and colleagues:

1. Respect for privacy vs vigilance in risk monitoring.
2. Differentiating normal vs risky behavioural change.
3. Familiarity vs anonymity in risk disclosure.
4. Respecting autonomy vs imposing constraints to limit risk.

A fifth tension operated between individuals and the broader service system:

- Dependence on vs perceived failures of community services.

### 3.3 Individuals vs their support system

#### 3.3.1 Tension 1: respect for privacy vs vigilant risk monitoring

Accurately monitoring mood and risky behaviour was identified by the majority of family/friends as critical to supporting and managing mood problems and suicidality. However, the men tended to perceive frequent inquiries about mood as invasive, irritating, and patronising or, a challenge to their sense of self. For example, one man reported:

With my closest friends it was, ‘I don't want you to know how I feel’. I'm a dad of three kids and a husband. I've got a good job. I don't want you to know that I'm so sad that I cry at red lights. (Interviewee, male, 36)

For another, his sense of masculine pride underlined his need for privacy, which was further reflected by one focus group member (FGM) who observed:

…they tend to say they would be better if you weren't pestering them by trying to get in contact with their feelings or their emotions… (FGM Male, 48)

On the other hand, checking‐in by family/friends was necessary for noticing warning signs, especially in men who didn't communicate their feelings or tended to isolate themselves when becoming more depressed or irritable. For example, this particular family/friend described retrospectively recognising changes in the behaviour of a colleague who had died by suicide:

When I reviewed the death of one of my colleagues, one of the things was that he changed his behaviour. He was not known for returning phone calls within a reasonable time. He might take three or four days, but then he got to almost a week and a half, two weeks…and that was a change of behaviour. (FGM, Male, 50)

Several negative outcomes were reported to result from this tension between respecting the man's privacy vs the need to be vigilant in risk monitoring. Often, anxiety increased, due to perceived loss of control by the individual or carers, and rapport between them was damaged. Some men became more reclusive and less likely to disclose their thoughts or distress. Even when men were relatively open about their suicidal plans, awareness of on‐going risk tended to increase stress and anxiety within the relationship, especially if they were conscious of being a burden:

I've had so many hard times…there's a point where there's so much recurrence that you feel that you're putting them through it a lot. And that you're not making any headway yourself…it's just to shield them, to keep them away from it because I don't feel comfortable putting them in that position (Interviewee, Male, 28)

Individuals and focus group members suggested a number of potential solutions to address this tension. Regular monitoring was seen by the majority of participants to be essential, despite the additional stress it may generate. Negative impacts may be ameliorated, however, by listening without judgement, providing at‐risk men with information about support services outside the family, or, with consent, by sharing information with other people in contact with the individual.

#### 3.3.2 Tension 2: differentiating normal vs risky behavioural change

Both at‐risk men and family/friends agreed that an important part of monitoring risk involved accurately recognising changes in behaviour. However, a second tension related to the difficulty in differentiating non‐harmful behaviour change from change indicating mood disruption and increased risk of suicidality. For example, one man related mood disruptions and missed opportunities for somebody to check in:

…and I yell at someone and bump into somebody else on the way out, if the [person had] said, ‘gee, it's not like [name]’ that would've helped too, but nobody chased me down the corridor to the doorway to say, ‘[name], come back. I want to talk to you’. That would've helped. (Interviewee, Male, 60)

Yet friends and family reported an inability to recognise these instances for what they were:

The other thing that I found difficult was to work out what was normal teenage behaviour and what was actually locking himself away because of being down…’is that suicidal behaviour, or him being a teenager?’ (FGM, Male, 39)

Participants noted that this tension was particularly relevant for adolescents, who often engaged in riskier behaviour, were more irritable and emotionally reactive and sought greater autonomy and privacy. However, even among adult men, behaviour change was also affected by things other than suicidality, such as unrelated relationship breakdowns. Conversely, statements of suicidal ideation or intent may be misinterpreted or judged to be lacking in veracity by family and friends:

We didn't probably take it serious enough when he was asking for help. And it's a horrible thing to say, but we all work and we're all busy and you've all got your own life and at the time I was just, you know, I'm busy, leave me alone. I shouldn't say that but I did. I felt guilt for a very long time after that I didn't give him more attention. (FGM, Female, 55)

Again, family/friends reported that the difficulties of accurately evaluating behavioural changes often led to either “false positives” contributing to conflict or “false negatives” resulting in insufficient support and self‐harm. Conversely, men acknowledged an inability to clearly communicate the risk:

It's one of those friction points, isn't it? Yeah. ‘Cause in a way I'm sabotaging my own ability to get better. And they're trying to find this way of helping me. And I'm just not helping. (Interviewee, Male, 53)

They highlighted the importance of consultation with general practitioners, clinical psychologists or counsellors, to decrease risk and anxiety associated with this tension.

#### 3.3.3 Tension 3: familiarity vs anonymity in risk monitoring

The relative benefit of risk assessment and monitoring carried out by people familiar to the man at risk, vs by independent individuals, such as health professionals, also emerged as a tension. On the one hand, familiar individuals were often better able to recognise and interpret idiosyncratic changes in behaviour:

I was aware that that wasn't his standard way of living, and it became an obvious sign. I do understand that it was only, though, because I actually knew the person for a longer period of time. Whereas… earlier on in that friendship or at an acquaintance level you wouldn't have the same understanding… You would just assume them to be a joker or a larrikin. (FGM, Male, 26)

However, in some instances, greater familiarity also made listening without judgement more difficult and reduced the likelihood of disclosure of important information. This was particularly true for those men who prioritised independent problem‐solving or did not want to be perceived differently after disclosing “weaknesses.” An independent person on the other hand could provide a different point of view, act as a “circuit breaker,” or bring clarity based on professional detachment. Anonymity and perceived freedom from judgement made it easier for men to open up about problems and feelings:

And I remember breaking down in the doctor's surgery. I was there just for an annual check‐up and as soon as he closed the door I was a mess…I wouldn't allow myself to show it to friends and family. It was to a stranger where it was kind of like you felt that if you were going to be judged it would be far less than what it would be from family and friends. (Interviewee, Male, 36)

However, at the same time, outside observers were less likely to identify idiosyncratic behavioural cues, signs of deception or behavioural change.

Some participants reported that this tension resulted in increased stress and perceived loss of control for parties, as well as ineffective risk monitoring and management. Simply choosing the wrong time to approach men, for example, could damage rapport or lead to inaccurate risk assessment, despite some men expressing a desire for others to notice changes in their behaviour or demeanour. One solution suggested by participants was to ensure that individuals are aware of and linked into both familiar and independent support systems during times of distress.

#### 3.3.4 Tension 4: respecting autonomy vs imposing constraints

A majority of participants identified managing risky behaviour as another critical aspect of supporting at‐risk men—as distinct from risk monitoring. The extent to which a man's autonomy was respected or constrained during this process represented a fourth complexity to be navigated. On one hand, family and friends reported that challenging his unhelpful thoughts and restricting his behaviour was often essential to keeping a man alive and safe, such as when intent to self‐harm was active and strong. However, removing a man's freedom to choose could put strain on relationships, trigger blame and distress and cause conflict.

Family/friends reported that men were often difficult to reach, describing various instances in which men expressed reluctance to engage with support, or refused to accept the type of care being offered. For example:

I know from my own personal experience with my dad, he won't accept the help really. I could set up a hundred different things, to be honest, but he'll say, no, I don't need it… There was no way I could make him even go and see his GP. So, it's a struggle when they put the wall up…he just kept saying, ‘no, no, no’ all the time. (FGM, Female, 57)

In addition, managing risk sometimes required family, friends or services to impose limits on men's choices in order to prevent harm, for example, restricting movement or access to potentially harmful materials or involuntary placement in inpatient health services. One participant described observing police intervene with a suicidal man:

…by the time I got down there, they've already got him off the side of the road… they got him in the police car and took him home. And he actually took a few swipes at the copper. A good guy, he just let go. Trying to help him was really hard. (Interviewee, Male, 29)

Similarly, several of the men argued that decision‐making by acutely suicidal men was often affected by impulsiveness, and lack of systematic reasoning or insight into other available options (such as accessing treatment or contact with support groups). As such, the majority of all participants agreed that managing risk of suicide sometimes required acting against the immediate wishes of individuals. Several participants argued that support should nevertheless attempt to improve perceived self‐efficacy by minimising unnecessary behavioural restrictions.

### 3.4 Individuals vs the service system

#### 3.4.1 Tension 5: dependence on vs perceived failures of community services

A final tension concerned the role of external services in managing risk of further suicide attempts. A majority of men and focus group participants expressed frustration at or criticism of welfare or health services supporting individuals at risk. However, participants also identified that services often had capacity to manage risk in ways not available within normal family and social networks.

Participants described various perceived failures of health or other services related to: assessment of mood disorder and suicidality, scope or quality of intervention, and the extent and clarity of communication with family members. Participants reported that these failures tended to damage relationships and faith in services, making on‐going support more difficult. One family/friend participant recounted how a failed intervention amplified their family member's hopelessness:

I took him to the doctor and they sent him to mental health, who put him in hospital to try and dry him out, which was a disaster. They ended up calling the police and they threw him down the hallway and handcuffed him, and he couldn't cope. He didn't get the right sort of help … And, that made him a lot worse. Then he just decided that he didn't want help anymore, that he would be fine, everybody just leave him alone… And he just kept escalating down, down, down. One bad experience is perceived as, ‘well, you are all the same’ and will lead to not seeking help again, almost suicide just to spite them. (FGM, Female, 55)

On the other hand, participants reported that health services sometimes played a critical role in supporting individuals at risk. This was due in part to their capacity to manage and contain risk more directly than family or friends, often indirectly helping supporters to deal with the stress.

I suppose I used more of what was actually out there than a lot of people did. A lot of people don't know what services are out there for those sort of things… psychologists, psychiatrists, counsellors, they're great, especially in a mental health plan. (Interviewee, Male, 18)

I thought I've got nothing to lose, I'll give this [psychologist] a go. There's some sense in what these guys are saying and they're not counsellors, they're not trying to tell me to smile and be happy. They're showing me the mechanisms of what's going on and I saw some sense in that, that we could change this… (Interviewee Male,38)

At times, simply changing the environment around a man acted as another useful “circuit breaker,” for example by generating a community around otherwise isolated individuals. This may be particularly important when supporting those who had alienated themselves through aggression, substance abuse or other avoidant behaviours.

Participants observed that frustration, confusion and distrust directed at the service systems tended to reduce the effectiveness of co‐operation between care providers and thereby the quality and consistency of support for individuals at risk. Several focus group members suggested that providing skills training and psycho‐education to families and friends early in interventions would improve support to individuals at risk, as well as provide an understanding of how service systems operate, reducing reliance on and resentment towards relevant services.

## 4 DISCUSSION

The tensions identified here had several negative impacts. Not only did they contribute to greater difficulty in family/friends identifying early warning signs of an impending suicide attempt, they also had the effect of limiting requests for help by suicidal men. However, the findings point to suggestions for improving interventions addressing suicidal behaviours in men and for enhancing health‐care providers’ approaches to working with affected family and friends. In particular, three areas emerged as targets for change: first, inadequacies in the way individuals or supports communicate risk information, behaviour or motives. Second, the lack of understanding, by both men and family/friends, of the processes, risk factors or warning signs. Third, the lack of flexibility in the delivery of support, by either family/friends or services, in response to the needs or preferences of individuals (eg, confidentiality).

Approaches to managing these tensions and for enhancing existing clinical interventions may need to incorporate the qualitative results presented here, as previously advocated.^28^ In particular, previous research^29^ identified the importance of a desire for control among men who have attempted suicide, which concords with these findings, and represents an area to focus on for future prevention.

### 4.1 Improving risk communication

Factors limiting men's willingness or skills to disclose important risk‐related information may be assisted by improving ease of risk reporting and asking for help, as well as men's self‐care skills. For example, individuals, family and friends, and indeed the public at large would benefit from psycho‐education about available support services within the community and effective self‐care strategies, alongside information about the tensions that might be expected in the course of managing suicide risk. Men would benefit from understanding alternatives for reporting distress or suicidal thinking, while at the same time keeping in mind personal preferences about confidentiality.

While not specifically suggested by the majority of men, it may be worth considering a more resource‐intensive process, which involves adapting or creating specialised services for male suicide support, alongside existing but indirectly related support networks, such as Men's Sheds.^30^ Given the tension reported by family/friends in understanding normal vs risky behaviour change, the men's desire for autonomy and the well‐established fact that men report lower rates of help‐seeking than women for suicidality,^31^ this approach has important benefits in reaching men in environments they already participate in. This is especially true for those men who wish to cope in a manner consistent with masculine values such as self‐sufficiency, problem‐solving and independence or those men who do not (at present) have the capacity to directly challenge culturally entrenched expectations about male behaviour. It is unclear from existing research whether tailoring service delivery specifically for men would significantly alter the rate of male suicide. It might be argued it is more important to increase service uptake among those men who have never sought care, at an earlier point in their illness, and better early‐detection of mental health issues for those men who consult health practitioners for other reasons. However, these preliminary results suggest that for some men who do already access services, they would benefit from receiving care that takes into account differences in their presentation and preferred strategies.^32^

Likewise, there is a need to raise awareness about the tensions identified here in existing public education campaigns that go beyond just at‐risk men and their family/friends, particularly with regard to promoting the message that these tensions are to be expected yet are surmountable. This concords with recent research that suggests a more nuanced understanding of how men access care, with a specific emphasis on the notion that responsibility for accessing services should not rest solely on a suicidal individual.^33^

### 4.2 Reducing reliance on single source supports

Both participant groups consistently reported that reliance on any one individual or group for risk monitoring increased systemic anxiety and risk, particularly given men's reluctance to seek help. This was particularly evident in tensions 1, 2 and 3, where men and their family and friends struggled with monitoring risk, over‐stepping boundaries, understanding what constituted an agreed threshold for action, and what might happen when service use was initiated. However, this concern appeared to be lower in cases where support was effectively integrated, such as through sharing information, whether amongst family members or health‐care professionals, and where loss of status was not perceived to be at stake, due to lack of judgement on behalf of the support person.

Skills training, consultation or psycho‐education about how service systems operate for men may reduce reluctance to access resources and increase ease of access, which may be particularly valuable if accessed by men during an earlier phase of distress. Use of online self‐help programmes on suicide prevention and/or men's mental health such as the myCompass programme^34^ may also be beneficial, especially for men who are concerned about stigma. Likewise, education for family/friends and clinical services that emphasises the existence and availability of such programmes could increase the range of resources available to all participants in the system. In addition, open communication about decision‐making processes by service system professionals, while in the short‐term more labour intensive, may also carry additional benefits in improving skills of services users, de‐centralising responsibility and care.

### 4.3 Increasing flexibility of intervention in response to individual needs

Managing risk may at times require acting against the explicit choices of individuals; however, our participants suggested that the effectiveness of interventions may be maximised by avoiding unnecessary restriction, involving men as much as possible in decision making and flexibly adapting to individuals’ preferences.^33^

Taking some responsibility for choosing to live, or working to change unhelpful behaviours, rather than maintaining total reliance on external forces to prevent men from hurting themselves was also judged to be essential. Providing this message to at‐risk men may help them to change unhelpful thinking patterns, especially when offered in the context of continued support from family/friends, belonging and connectedness. However, if pressured inappropriately, it may add to their feelings of distress or incompetence.

Conversely, men may choose to take responsibility for their care, in which case, effort placed on “skilling up” men with regard to self‐regulation and coping may allow them to address problems with independence from support networks, at the same time enhancing self‐esteem, self‐efficacy and promoting long‐term change. It appears important to access available resources within care systems flexibly or adaptively, considering specific risks, needs and preferences within the system.

### 4.4 Limitations

The study is potentially limited in that a majority of the sample were aged 40 years or more, there were no men aged over 67 years, and a majority were not in current partnerships. Australian data^9^ show that men over the age of 80 years have a higher rate of suicide than young men, with men aged 85 years or more accounting for the highest suicide rate. It may be the case that important perspectives of younger men, older men with higher suicide risk, or those men at high risk of suicide involved in intimate partnerships, are excluded from the study. This could be particularly important given the high mental health burden experienced by younger age groups, or the potentially health buffering effects of partnerships. Future research should seek to clarify whether different tensions in suicide prevention exist among younger age groups, the factors affecting the increased rate of suicide in older men, and whether the retrospective reflections of middle‐aged men, as presented here, represent perspectives that could be usefully applied in an early intervention context.

## 5 CONCLUSIONS

Previous research has shown that there are considerable gender differences in suicidal risks and behaviour.^6^ Our results suggest that development of suicidal behaviours in men may also be complicated and compounded by tensions between men and their support network. Ways for improving suicide interventions for men were also identified. Specifically, communication of risk information and responsibility for care needs to be spread across systemic resources to reduce concentration and mitigate risk. In addition, opportunities for decision making, self‐efficacy and personal responsibility need to be maximised, while interventions consider and adapt to the idiosyncratic needs of individuals.
